# Antipsychotics, COVID-19, and Secondary Healthcare Databases: Revisiting the Pandemic

**DOI:** 10.1093/ijnp/pyae026

**Published:** 2024-06-15

**Authors:** Xavier Boland, Luiz Dratcu

**Affiliations:** Lewisham Hospital, South London and Maudsley NHS Foundation Trust, Denmark Hill, London, United Kingdom; Maudsley Hospital, South London and Maudsley NHS Foundation Trust, Denmark Hill, London, United Kingdom

**Keywords:** Antipsychotics, psychotic disorders, acute inpatient psychiatry, COVID-19, electronic medical records

Over the last 2 decades, the digitization of much of healthcare provision has seen a burgeoning of electronic medical record (EMR) databases. The ready availability of large-volume clinical data, at low cost, has opened new opportunities for epidemiological research. Yet issues of data quality and suitability for research purposes can compromise the task of translating EMR data into reliable clinical evidence (Parums, 2021; Honeyford et al., 2022).

The controversy around the safety of antipsychotics during the COVID-19 pandemic illustrates how potentially misleading outcomes of some such studies may adversely affect clinical practice. Addressing this critical topic, the study by Pintos-Rodríguez et al. (2024) raises highly pertinent questions regarding the credibility of large epidemiological studies that are entirely based on data obtained from secondary EMR databases but which fail to adopt adequate methodological safeguards.

## Prescribing Antipsychotics and COVID-19: Databank Research

Epidemiological findings early in the COVID-19 outbreak had shown preexisting severe mental illness (SMI) to be associated with increased risks of contracting, developing complications to, and dying from COVID-19 (Lee et al., 2020; Fond et al., 2021; Hassan et al., 2022). This prompted a discussion on the contribution of antipsychotic use to excess morbidity and mortality from COVID-19 in patients with SMI.

Thus, commenting on a retrospective cohort study published by Yang et al. in Lancet Healthy Living (2020), which used UK Biobank records to test the association between preexisting psychiatric diagnosis and COVID-19 outcomes, Ferraris et al. (2021) argued that using antipsychotics could increase vulnerability to COVID-19 complications and hence explain the worse outcomes seen in patients with SMI. Yet the analysis by Yang et al. did not include any information on psychotropic medication received by patients. Later, a meta-analysis by Vai et al. (2021) of 3 studies using clinical data of 22 613 patients derived from regional or national consolidated EMR databases found a strong positive association between exposure to antipsychotics and COVID-19 mortality (odds ratio 3·71, 95% CI:1·74–7·91). From this, Vai et al. inferred antipsychotics may precipitate cardiovascular risk in COVID-19, disrupt immune function, or interact with drugs used in the treatment of COVID-19.

Ferraris et al. and Vai et al. stopped short of advocating changes to clinical practice. For practicing psychiatrists, however, this might have been enough reason for disquiet. Had these warnings percolated clinical services and led to changes in prescribing guidance during the pandemic, the consequences could have been disastrous. At a time of crisis, and in the absence of countervailing epidemiological evidence, there might have been calls to discontinue antipsychotic treatment in patients who had been receiving it and a new reluctance to start antipsychotic treatment in those who needed it. Worryingly, these warnings were almost coetaneous with claims that antipsychotics are no more than sedating agents that cause “mental suppression” (*sic*) and that relapse following treatment discontinuation may be just antipsychotic withdrawal effects (Horowitz et al., 2022). All services caring for patients with SMI, particularly acute inpatient services, could thus find themselves facing a major dilemma. After all, how could well-meaning clinicians possibly prescribe such drugs to patients with SMI, especially during the COVID-19 pandemic?

As it turned out, reanalysis of the data by Yang et al. (2021) concluded that the use of antipsychotics had little impact on COVID-19 outcomes, if any. Similarly, further scrutiny of the studies included in the meta-analysis by Vai et al. found that their results were highly unlikely to reflect the profile of most patients with SMI receiving antipsychotics. As we pointed out at the time, the study by Vai et al. had methodological shortcomings that compromised their findings. Chief among these was the inclusion of working-age patients with primary psychotic or mood disorders and older age patients suffering from dementia and medical comorbidities in single cohorts, despite their markedly different clinical profiles and underlying vulnerabilities to COVID-19 (Boland and Dratcu, 2021). Pintos-Rodríguez et al. (2024) have demonstrated that, when these shortcomings are addressed, evidence actually shows antipsychotics do not increase COVID-19–associated risks.

## Prescribing Antipsychotics and COVID-19: The Clinical Setting

In fact, reports arising from the inpatient setting suggested that those patients receiving antipsychotic treatment consistently had fared surprisingly well during the pandemic (Dratcu and Boland, 2020; Boland and Dratcu, 2020a, b). Some clinical studies have lent support to these observations. A Spanish retrospective cohort study of 698 patients with schizophrenia receiving long-acting antipsychotics, with at least 80% compliance, found that patients actually had a lower risk of infection and better outcomes after contracting COVID-19 compared with the general population (Canal-Rivero et al., 2021). In an Argentinian comparison of 121 hospitalized COVID-19–positive patients with schizophrenia or bipolar disorder with a matched cohort of COVID-19–negative patients subject to the same exposure to the SARS-CoV-2 virus, those receiving clozapine were 60% less likely to contract COVID-19 (Prokopez et al., 2021). Also, a cohort study of 1958 inpatients with SMI in New York state psychiatric hospitals who had undergone COVID-19 testing found that the use of second-generation antipsychotics was associated with a marked reduction of the risk of COVID-19 infection (odds ratio = 0.62; 95% CI = .45–0.86) (Nemani et al., 2022).

Unlike studies of samples obtained from large electronic databases, patients recruited to treatment cohorts in each of these smaller studies were all identified as having a formal psychiatric diagnosis, thus establishing a clear indication for antipsychotic treatment. Moreover, steps were taken in each instance to ensure patients included in the analysis were adherent to treatment.

Unless specific criteria are used, scrutiny of data from EMRs might not always reflect the complex needs of patients with SMI. There is in fact good reason to suggest that, rather than increasing risk for patients with SMI, antipsychotics may actually protect them from COVID-19. First, effective symptom control with antipsychotics is likely to curtail many of the vulnerabilities to COVID-19 associated with untreated SMI. Second, there is emerging evidence that antipsychotics have anti-inflammatory properties that may not only play a part in the treatment of SMI itself (Mondelli and Howes, 2014) but also confer direct protection by hindering cytokine storms that underlie COVID-19 complications (Dratcu and Boland, 2021).

Obviously, antipsychotics are not devoid of adverse effects. Of note, metabolic side-effects of some antipsychotics have been associated with a significantly higher risk of obesity and diabetes in patients with SMI (Vinogradova et al., 2010; Holt, 2019). Both are major vulnerability factors for poor COVID-19 outcomes and could be expected to contribute to an increased risk of COVID-19 complications in this patient group. That this has not been observed in SMI patients receiving antipsychotics lends further support to the notion of an overall protective effect of antipsychotics in COVID-19.

## Antipsychotics, The COVID-19 Pandemic, and Beyond

The interplay between SMI, antipsychotics, and COVID-19 has important implications for clinical practice as it will inform decisions to initiate, continue, or stop what is often essential treatment for patients with SMI. An overreliance on epidemiological research derived from EMR data, at the expense of clinical observations or experimental and clinical research, can limit the ability to answer complex clinical questions. Indeed, there is still no consensus as to how best to assess data fidelity and accuracy of electronic health records when used in research (Cowie et al., 2017). Consequently, unless researchers adopt strict methodological criteria specific to the questions they seek to answer, retrospective epidemiological studies using such data will produce results that can ultimately prove misleading, with detrimental consequences to patient care. The study by Pintos-Rodríguez et al. proves this point.

Antipsychotics are not perfect and are not all the same, nor are they always used as they should be. Research failing to account for this is bound to expose itself to criticism, which ideally should happen before controversial outcomes lead to misguided changes in patient care. In psychiatry, the risk of unwelcome consequences, such as withholding treatment, cannot be overestimated, particularly in the case of antipsychotic use. Similarly, poor adherence to antipsychotic treatment, itself a measure of patient confidence in treatment, remains the main reason for relapse and readmission to hospital in patients with SMI.

If ever there was a pressing need to educate patients and convince reluctant prescribers about the importance of adherence to antipsychotic treatment, and of commencing it in those who need it, the pandemic has now made it imperative. There is at this stage no shortage of evidence supporting the safety and effectiveness of antipsychotics when properly prescribed. It is up to practicing psychiatrists to reassure their patients that, COVID-19 or not, antipsychotics are mostly safe and effective, in the knowledge that the evidence for good practice is fully on their side.

## Data Availability

No new data were generated or analyzed in support of this research.
